# Optic Disc Drusen (ODD), an Often Misdiagnosed Disease: A Case Report

**DOI:** 10.7759/cureus.32664

**Published:** 2022-12-18

**Authors:** Mohamed Bouazza, Amine Razzak, Mehdi El Jai, Houda Youssefi

**Affiliations:** 1 Department of Ophthalmology, Faculty of Medicine, Mohammed VI University of Health Sciences (UM6SS), Casablanca, MAR

**Keywords:** optic neuritis, optic disc drusen, visual field loss, papilledema, optical coherence tomography

## Abstract

Optic disc drusen (ODD) are abnormal collections of protein and calcium that accumulate within the optic nerve. We report a case of a 17-year-old girl who presented to the Department of Ophthalmology at the Cheikh Khalifa International University Hospital, Casablanca, Morocco, with a decline in visual acuity, visual field deficiency, and color vision abnormalities. The patient was misdiagnosed and mistreated for optic neuritis given the presence of bilateral Stage III papilledema solely. After many months of diagnostic wandering, a fundus examination revealed a white atrophic papilla with calcified deposits grouped in a crown around the papillary excavation, suggesting papillary drusen. When the deposits are visible on ophthalmoscopy and manifest as an elevation and a blurring of the optic disc's margins, their diagnosis remains straightforward. However, their identification might be problematic when they are firmly lodged in the optic disc or with the presence of papilledema, leading to confusion with other differential diagnoses, particularly if the condition affects both eyes. The purpose of this case report is to increase neurologists' and ophthalmologists' knowledge of the incidence of drusen in order to prevent excessive biological and imaging investigation in addition to harmful effects from needless drugs.

## Introduction

Optic disc drusen (ODD) are aberrant accumulations of calcified mitochondrial deposits at the head of the optic nerve [1]. Our knowledge of drusen has advanced significantly during the last decade with the advent of high-resolution in vivo retinal imaging. Typically, the diagnosis is discovered by coincidence during a routine medical examination. ODD is also asymptomatic due to the rarity of visual loss. Nevertheless, patients might present complications including anterior ischemic optic neuropathy, retinal vascular occlusions, and peripapillary choroidal neovascular membranes (CNV) that might prompt the patient to consult [2]. In contrast, peripheral visual field loss is far more prevalent. Both congestive papillae and optic disc atrophy might be mistaken for drusen papillae. The differentiation from congestive papillae is crucial [3]. Misdiagnosis may, therefore, lead to enormous expenses in terms of paraclinical explorations and important long-term therapy-related side effects. The diagnosis of papillary drusen is straightforward when the deposits are apparent on ophthalmoscopy and present as an elevation and a blurring of the optic disc's margins. However, their identification might be problematic when they are firmly lodged in the optic disc, leading to confusion with papilledema, particularly if the condition affects both eyes. A subset of criteria based on multiple imaging modalities and microscopic appearance and molecular composition is used to identify and categorize ODD. It is noteworthy that optical coherence tomography (OCT) is the gold standard in diagnosing and staging ODD. Severe visual impairment secondary to ODD is very uncommon and the latter has a favorable prognosis overall [4]. To date, surgical excision or decompression as well as pharmaceutical treatments were generally deemed unsuccessful.

## Case presentation

**Figure 1:**
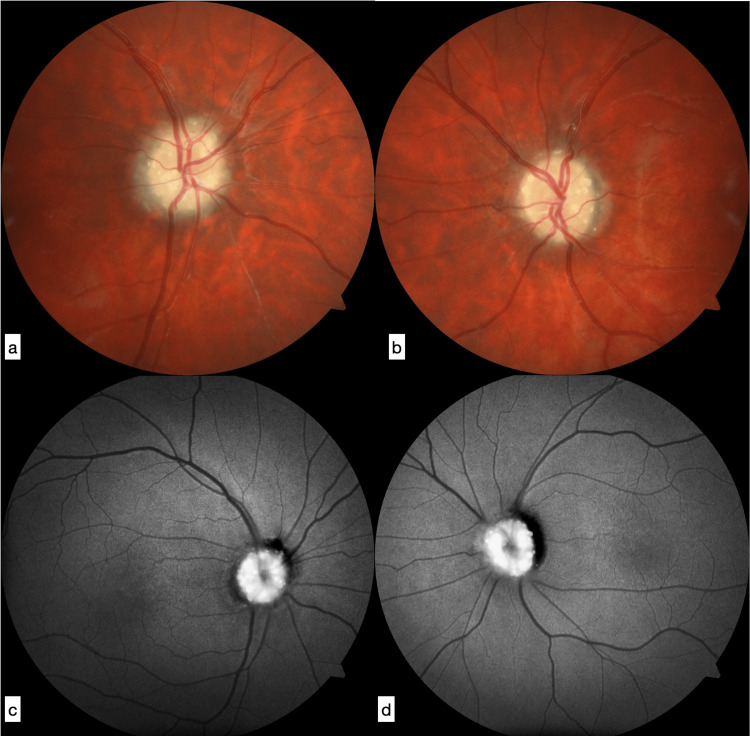
Retinography showing a white atrophic papilla with calcified deposits grouped in a crown around the papillary excavation, suggesting papillary drusen: (a) right eye, (b) left eye. FAF imaging showing several hyperautofluorescent lesions with irregular borders in the papillae: (c) right eye, (d) left eye. FAF: Fundus autofluorescence

A 17-year-old girl was initially admitted to Cheikh Khalifa International University Hospital, Casablanca, Morocco, with a prior history of bilateral inflammatory arthralgias in the hands, wrists, ankles, and knees for many months. Her family history was notable for a mother and an uncle who were followed for Still's disease and sarcoidosis, respectively. Several months before admission, the patient reported a decline in visual acuity, a visual field deficiency, and color vision abnormalities. The patient was diagnosed with bilateral Stage III papilledema after the first ophthalmological examination conducted a year ago. Her papilledema was deemed secondary to inflammatory optic neuropathy even though her blood tests, brain MRI, and lumbar puncture were normal. The patient was given two cycles of methylprednisolone, receiving continuously on D1-D3 1g/day which greatly improved her symptoms. During the follow-up, the patient received oral corticosteroids and azathioprine (Imurel) simultaneously. Six months later, as part of her routine neurological follow up, the patient's ophthalmological examination revealed 6/10 visual acuity in the left eye and 9/10 in the right eye. On the fundus, a white atrophic papillae with calcified deposits grouped in a crown around the papillary excavation, suggesting papillary drusen. The anterior segment examination was normal and autofluorescence retinography showed several hyper autofluorescence lesions with irregular borders in both papillae (Figure 1).

**Figure 2:**
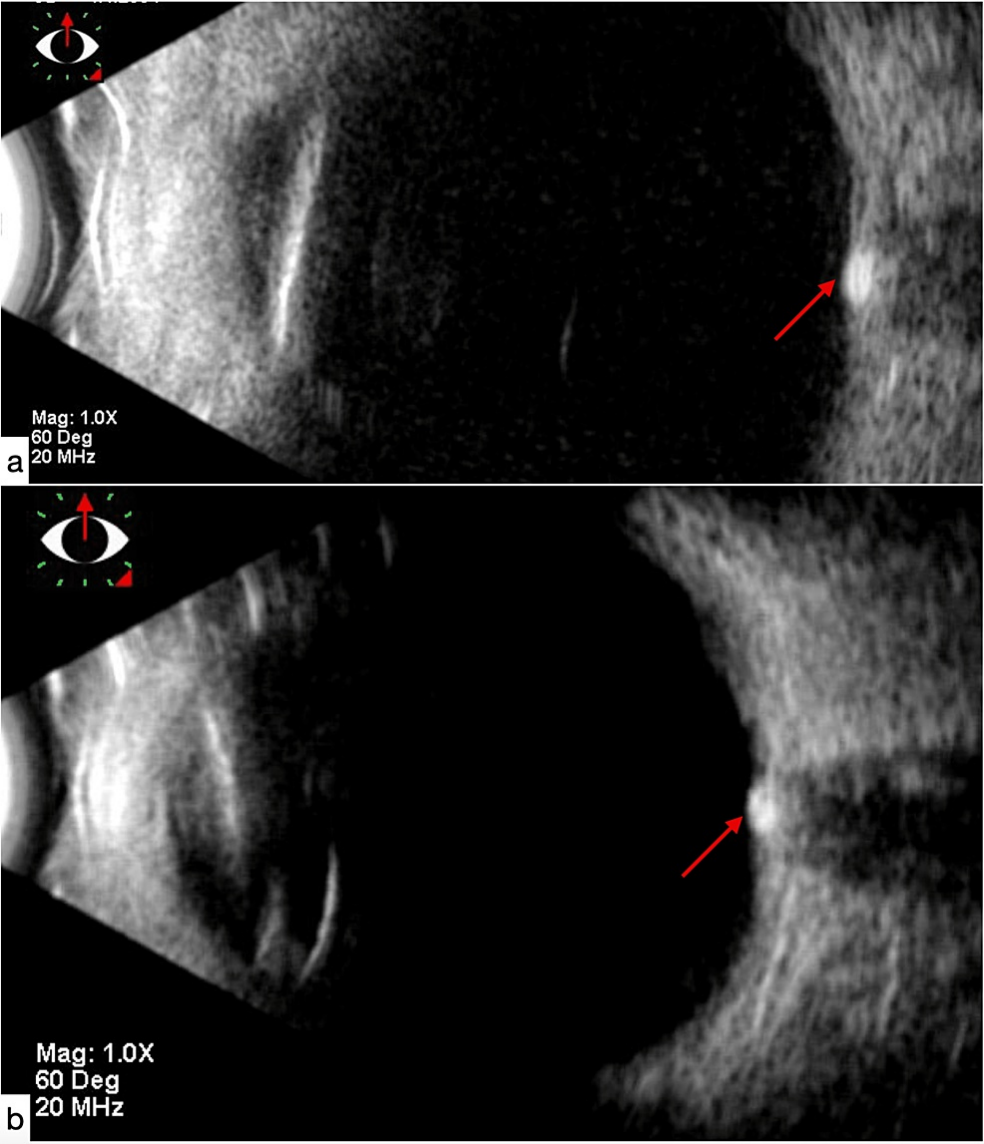
Hyperechogenic appearance of papillary drusen (red arrow) on B-mode ultrasound : (a) right eye, (b) left eye.

Hyperechogenic calcification with a posterior shadow cone is seen at the optic nerve head on the ocular ultrasound (Figure 2).

**Figure 3:**
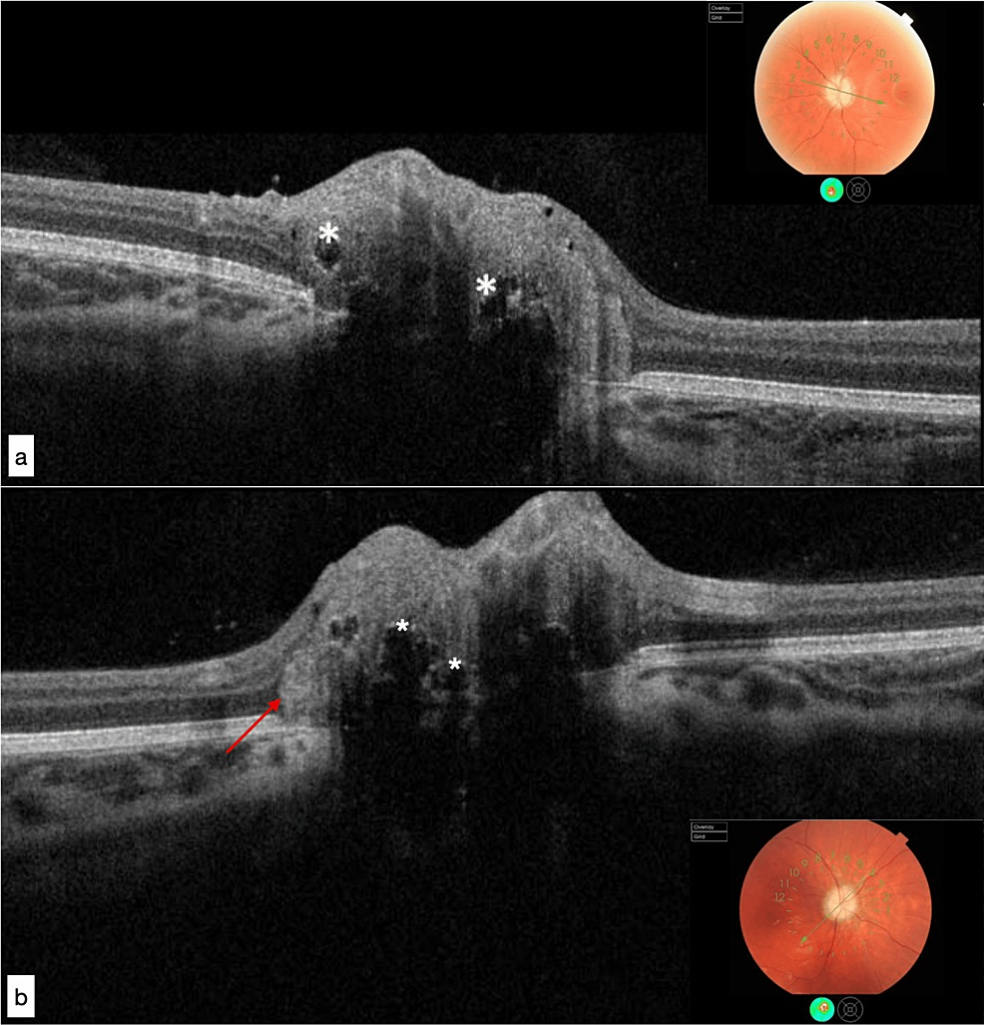
Papillary OCT showing numerous small- to medium-sized superficial ODDs (white asterisks) with a bubble-like appearance encompassing the entire optic disc and PHOMS (red arrow): (a) right eye, (b) left eye. OCT: Optical coherence tomography; ODD: Optic disc drusen; PHOMS: Peripapillary hyperreflective ovoid mass-like structures

Multiple optically empty agglomerated voids bordered by hyperreflective edges were identified using papillary OCT (Figure 3).

**Figure 4:**
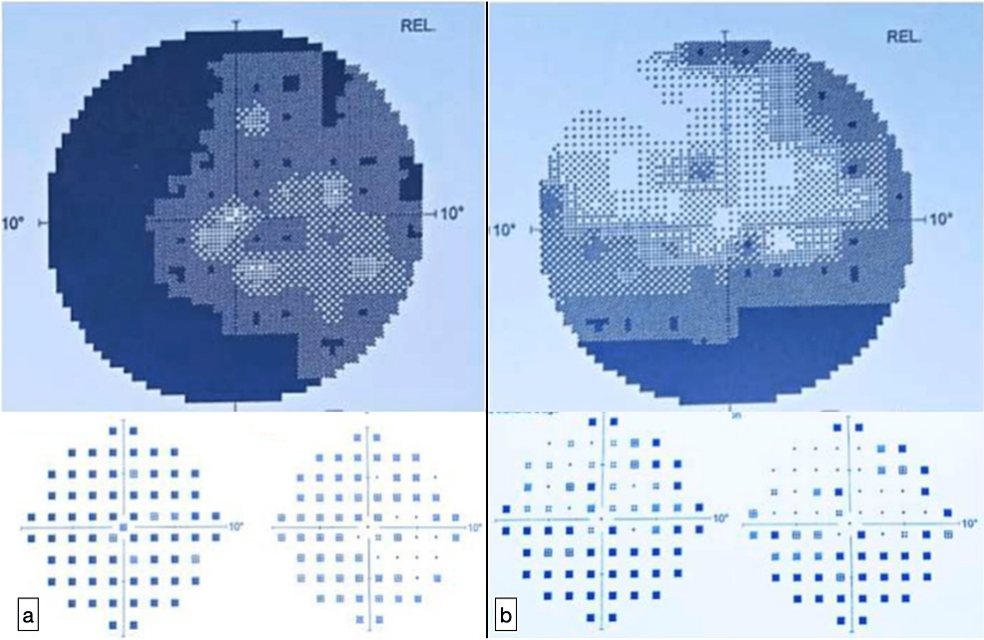
Severe visual field alteration : (a) right eye, (b) left eye.

The visual field examination showed deep and diffuse deficits in both eyes (Figure 4).

## Discussion

Papillary drusen are acellular deposits in the optic nerve head. Their frequency in the general population is predicted to be between 0.3% and 2.4% [5]. A narrow scleral canal is believed to generate axonal distress by a crowding effect that limits the axonal transit capacity of neurons, leading to axon degeneration and ODD development. The second explanation of drusen development postulates that ODD is caused by aberrant vasculature of the optic disc, producing axonal ischemia, distress, and poor axonal metabolism, which leads to drusen formation [6]. This is indicated by the relationship between drusen and aberrant optic disc vasculature observed during the examination, as well as decreased blood flow in the central retinal artery as evaluated by color Doppler imaging. It is hypothesized that calcium metabolism is involved in the production of drusen in response to axonal injury [7]. Drusen pathogenesis revealed histologically aberrant axonal metabolism: calcium, mucopolysaccharides, and nucleic acids are almost all constituents that escape from mitochondria into extracellular spaces. Although often asymptomatic, papillary drusen are linked with more or less significant visual field abnormalities in more than 50 to 90% of cases, due to the main injury to the optic fibers as well as their compression by massive calcifications in a limited scleral canal [8]. Moreover, according to longitudinal study research demonstrates that drusen often increase in size with time, especially in the growing phase, and often as a result the visual field deteriorates rapidly throughout adolescence, becoming more noticeable in the first two or three decades, before regressing [9].

Similarly, our patient featured a broad and significant bilateral visual field impairment, and her symptomatology has progressively deteriorated over the last two years, becoming very detrimental to her daily life and access to education, requiring constant coordinated support from her family. In these cases, misdiagnosing ODD as papilloedema may wrongly induce extensive neuroradiological imaging in order to explore the etiologies of intracranial hypertension. Our patient had initially undergone neuroradiological imaging in order to explore the etiologies of intracranial hypertension and was misdiagnosed with inflammatory optic neuropathy. The patient was also subjected to arduous medical therapy with significant adverse effects. Given that papilloedema is frequently bilateral in affected individuals, it may be challenging to detect and distinguish ODD when they are buried within the optic nerve head tissue. Because of this, many patients with buried ODD go through time-consuming, unnecessary investigations to rule out other potential causes of elevated intracranial pressure. OCT, which is now the gold standard for detecting both buried and superficial forms of drusen, can be used to diagnose ODD patients, potentially saving them from invasive testing.

Furthermore, improvement of the patient's visual acuity can be explained by the anti-inflammatory and anti-edema effects of corticosteroids. Both optic disc edema and inflammation were a result of the drusen’s substantial compression on ganglion cell axons. After one year, the follow-up examination revealed stable visual acuity and visual field. Therefore, topical treatment based on beta-blockers and citicoline was maintained in order to slow down the progression of ganglion cell loss.

Historically, a fundoscopic examination or imaging has been used to diagnose ODD in vivo. Similarly to this case, ODD appears on OCT as variably sized masses with a signal-poor core and hyperreflective anterior margins of various sizes in the unmyelinated optic nerve. Both peripapillary hyperreflective ovoid mass-like structures (PHOMS), which represent the lateral bulging of dysmorphic axons, and horizontal hyperreflective lines, which may represent ODD precursors, are frequently found as represented in our case. In the early stages of the disease, the papillary OCT is useful for evaluating the retinal nerve fiber layer (RNFL). The disc in our case was completely atrophic, so the OCT cannot provide accurate measurements. ODD may occasionally not be visible, especially in children, making the diagnosis of ODD using only fundoscopic examination or imaging challenging. To date, the Optic Disc Drusen Studies Consortium has recommended enhanced depth imaging optical coherence tomography (EDI-OCT) as the gold standard for ODD diagnosis. Despite its sensitivity in the diagnosis of ODD, it takes time and training to review serial EDI-OCT B-scans in patients and identify different features, which is not always possible in a busy ophthalmologist's office. A promising method for diagnosing ODD is comprehensive multimodal ophthalmic imaging. In addition to EDI-OCT, fundus autofluorescence (FAF) and near-infrared reflectance (NIR) imaging are commonly used [10].

The therapy approach of drusen is entirely symptomatic. There is no known curative therapy and the previously advised decompression of the optic disc has never been shown to be effective in controlled studies. The same holds for "circulation-enhancing" and "neuroprotective" drugs [11]. ODD's variable consistency and hardness make them unpredictable, and there is currently no surgical codified treatment for optic disc drusen due to the proximity of functionally essential and sensitive structures, such as optic nerve fibers and retinal blood vessels, that must be handled with care [12]. Therefore, it is vital to develop ablating laser procedures for the safe removal of extremely hard materials [13]. Even though there is no curative treatment, as a precaution, patients with drusen papillae are often instructed to reduce their usual intraocular pressure [14]. Additionally, the therapeutic threshold will be set lower than for patients whose optic discs can be readily and reliably regulated. Therefore, our patient was administered a combination of beta-blockers eye drops, and topical citicoline in order to promote structural support for the repair of damaged cell membranes and enhance the bioelectrical responses of the retina (amplitude of the electroretinogram pattern) and the conduction of the optic pathways (amplitude and latency of the visual evoked potential) [15].

## Conclusions

This case exemplifies the medical complexity surrounding the diagnosis of papillary drusen. Therefore, to prevent needless and intrusive examinations and therapies, ophthalmologists and neurologists must be aware and invoke this diagnosis in the presence of any unlabeled or atypically developing papilledema. In these circumstances, papillary OCT is the gold standard for diagnosing ODD and should be performed upon any initial diagnostic ambiguity.
